# LEOPARD Syndrome with a Sporadic *PTPN11* Mutation in a Saudi Patient

**DOI:** 10.1155/2023/4161574

**Published:** 2023-05-23

**Authors:** Hussein M. Alshamrani, Luai M. Assaedi, Jumanah A. Bahattab, Abdulrahman M. Mohammad, Magdy R. Abdulghani

**Affiliations:** ^1^Department of Dermatology, King Abdulaziz University, Jeddah, Saudi Arabia; ^2^Department of Dermatology, King Abdulaziz Hospital, Mecca, Saudi Arabia; ^3^Department of Dermatology, King Fahd Armed Forces Hospital, Jeddah, Saudi Arabia; ^4^Department of Dermatology, Althaghr Hospital, Jeddah, Saudi Arabia

## Abstract

LEOPARD syndrome (LS) is a rare autosomal dominant inherited or sporadic genetic disorder caused commonly by missense mutations in the protein-tyrosine phosphatase-nonreceptor type 11 (*PTPN11*) gene. Due to its rarity and a high chance of misdiagnosis, the epidemiological profile of LS is poorly established. To the best of our knowledge, this is the second report with a documented *PTPN11* gene mutation in Saudi Arabia.

## 1. Introduction

LEOPARD syndrome (LS) is a rare autosomal dominant inherited or sporadic genetic disorder caused by missense mutations in the protein-tyrosine phosphatase-nonreceptor type 11 (*PTPN11*) gene [1]. The acronym LEOPARD stands for its multisystem clinical manifestations: lentigines, electrocardiogram conduction abnormalities, ocular hypertelorism, pulmonary stenosis, abnormal genitalia, retarded growth, and sensorineural deafness [2]. Due to its rarity and a high chance of misdiagnosis, the epidemiological profile of LS is poorly established. Here, we report a case of LS with a sporadic mutation in the *PTPN11* gene.

## 2. Case Presentation

A 5-year-old girl born to second-degree consanguineous parents presented with multiple lentigines that had begun to appear at the age of 2 years over her face, trunk, and thighs.

Notably, the mother had no follow-up with the obstetrician during the pregnancy and the patient was a full-term baby delivered through cesarean section, with normal weight and activity. All the previous pregnancies of the mother were normal with healthy children, and the family had no history of lentigines, congenital heart diseases, learning difficulties, speech delays, or syndromic disorders. A physical examination revealed the height and weight of the child to be 101 cm (3rd centile) and 13.9 kg (below the 3^rd^ centile), respectively, and the child had an occipitofrontal circumference of 52 cm. She showed facial dysmorphism, including a triangular face, slightly protruded mandible, broad nasal bridge, ocular hypertelorism, palpebral ptosis, low set ears, low posterior hairline, pectus carinatum, and scapula alata. No other skeletal anomalies were noted.

After birth, a pan-systolic murmur was detected. Three days after birth, an echocardiogram revealed multiple ventricular septal defects (VSDs). The patient has been continuously followed up with echocardiogram till date. At the age of 4 months, her mother started to notice that the baby was quiet and did not react to sound stimuli. An audiogram was performed at the age of 7 months and showed sensorineural hearing loss for which she underwent a cochlear implant at the age of 2 years. She also had delayed speech and motor development; she started crawling at 13 months and started to walk at 24 months.

**Figure 1:**
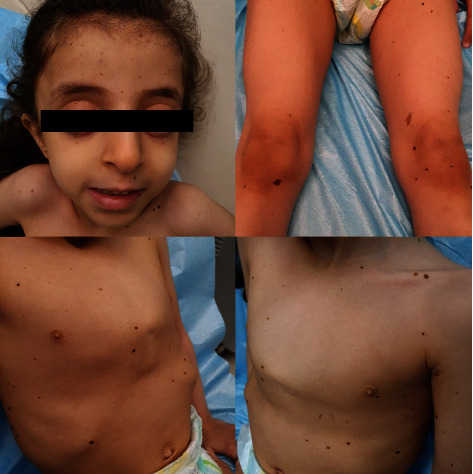
Multiple lentigines distributed on the face, trunk, and extremities of the pectus carinatum. Note the *cafe´ noir* spot and *cafe-au-lait* spot near the knee.

**Figure 2:**
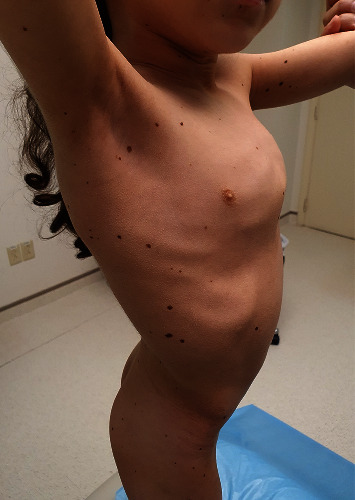
Multiple lentigines found distributed on the trunk of the patient on further physical examination.

At our hospital, the clinical examination of her lentigines showed multiple oval light brown macules, 2–4 mm in size, over both the sun-exposed and sun-protected body sites including trunk and upper and lower extremities (Figures 1 and 2). There was one darker lentigo, 5 mm in size (*café´ noir* spot) over the right knee, and a *café-au-lait* spots (brown patch), 1 cm in size, over the left thigh. Oral and genital mucosa were spared.

LS was suspected and the child underwent genetic testing, which showed a heterozygous mutation in *PTPN11*, c.836A > G, a missense variant resulting in the substitution of the tyrosine codon at amino acid position 279 with a cysteine codon (p.Tyr279Cys). LS was thus confirmed, and multidisciplinary follow-up was performed.

## 3. Discussion

To the best of our knowledge, this is the second report with a documented *PTPN11* gene mutation in Saudi Arabia, after the first one being reported by Alfurayh et al. [3] A comparison of the clinical features of the other cases reported in Saudi Arabia is shown in Table 1.

The negative parental and family history in our case indicates a possible sporadic form of LS that resulted from a new mutation, as shown in a recently published case report with a novel *PTPN11* mutation and negative genetic tests in the parents [4]. However, there still exists the possibility of nonpenetrance (one or two parents may have had the mutated gene but never developed the disease).

Since prenatal diagnosis is possible to be performed for LS-associated conditions as for other RASopathie [5], the age of diagnosis in our patient could also have been more precocious. Cardiac defects such as VSDs can be detected as early as at 16–18 weeks of gestation and genetic syndromes are usually linked with features such as increased nuchal transparency through ultrasound examination [6], which was not performed in our case. However, even with those parameters combined, the diagnosis of LS would have remained difficult to confirm without lentigines, the hallmark lesions of this syndrome.

To eliminate the possibility of nonpenetrance and detect causal mutations in the fetus, genetic testing is the best screening method for LS-associated mutations. To date, 12 missense *PTPN11* gene mutations associated with LS have been detected: Tyr279Cys/Ser, Ala461Thr/Ser, Gly464Ala, Thr468Met/Pro, Arg498Leu/Trp, Gln506Pro, and Gln510-Glu/Pro [1, 7]. It is, therefore, recommended to perform the sequence analysis of *PTPN11* gene for the coding exons 7, 12, and 13, which are known to be associated with LS. If this test is negative, sequence analysis should then involve LS-linked coding exons from *RAF1* and *BRAF* genes (6-13-16 and 6-11-17, respectively). In case no mutation is identified, the remaining coding exons of *PTPN11* as well as exons of other genes associated with LS, such as *RAF1* and *BRAF*, should be analyzed as well [1].

Besides the known characteristics of LS, our patient also had atypical cardiac findings like those of multiple VSDs without hypertrophic cardiomyopathy (HCM) or pulmonary stenosis. The absence of HCM may signify a favorable prognosis as the overall survival in LS is mainly conditioned by the severity of cardiac defects and this may support the hypothesis of the possible correlation between missense mutations and less serious forms of the disease [8]. However, since the possibility of developing HCM at a later stage still exists, the patient will have to be kept on long-term and stringent clinical and echocardiographic monitoring [9]. Furthermore, an oncology follow-up is considered due to the increased risk of tumor development similarly to those who are affected by dysregulation of the molecular mechanisms responsible for the control of cellular replication [10].

In conclusion, we presented a possible sporadic case of LS caused by a *PTPN11* gene mutation with typical clinical presentation except for the absence of HCM. Dermatological findings were crucial to suspect the diagnosis in our case. Therefore, we recommend physicians to be cautious when dealing with particular skin lesions (lentigines and *café-au-lait* spots), specifically when associated with pediatric age, and with facial dysmorphism as in other differential diagnosis [11], as this might be the first diagnostic indicator to uncover a serious potentially deadly condition. Due to advances in genetics and molecular biology such as microarray analysis [12], significant progress has been accomplished in the last decades in understanding many complex genetic disorders such as LS and other RASopathies. However, this progress has been substantially limited by challenges regarding the development of effective therapies. Further specific guidelines on the management of LS and the collection of more epidemiologic data are required. Success in the ongoing preclinical studies would allow current efforts to headway into the human trials phase, especially those of RAS pathway inhibitors and genome-modifying therapies.

## Figures and Tables

**Table 1:** Comparison of the clinical features of LS in the reported cases in Saudi Arabia.

	Our case	Case 1 of Alfurayh et al. [3]	Case 2 of Alfurayh et al. [3]	Case 3 of Alfurayh et al. [3]
Age/sex	5 years/female	37 years/male	4 years/male	18 months/male
Age of first lentigines	2 years	Unknown	6 months	2 months
Height (in cm) (percentile)	101 (3rd)	163	103 (50th)	88 (>95th)
Cardiac defects	VSD	ASD	−	ASD and VSD
Pulmonary stenosis	−	−	−	−
Deafness	+	−	−	−
Ocular hypertelorism	+	+	+	+
Pectus excavatum/carinatum	Pectus carinatum	Pectus excavatum	Pectus excavatum	−
Motor delay	+	+	+	+
*PTPN11* mutation	Exon 7, c.836A>G, p.Tyr279Cys	Exon 7, c.836A>G, p.Tyr279Cys	Exon 7, c.836A>G, p.Tyr279Cys	Exon 7, c.836A>G, p.Tyr279Cys

VSD, ventricular septal defects; ASD, atrial septal defects

## Data Availability

All data related to the article are available from corresponding authors upon request.
